# Special Issue “Molecular Mechanisms of Bisphenol A Toxicity and Effects of Environmental Levels on Health”

**DOI:** 10.3390/ijms24098028

**Published:** 2023-04-28

**Authors:** Lucia Coppola, Cinzia La Rocca

**Affiliations:** Center for Gender-Specific Medicine, Italian National Institute of Health, 00161 Rome, Italy; lucia.coppola@iss.it

Bisphenol A (BPA) is a plasticizer that is widely used in the manufacturing of polycarbonate plastics (PC) and epoxy resins for use in a broad range of consumer products, including materials in contact with food and beverages, as well as medical devices, toys and dental sealants. BPA has been characterized as an endocrine disruptor chemical (EDC) that is able to interact with and activate endocrine receptors that alter hormonal homeostasis. Indeed, BPA has been reported to interact with estrogen receptors (ERα and ERβ), androgen receptors (ARs), thyroid hormone receptors (TRα and TRβ), glucocorticoid receptors (GRs), non-canonic estrogen-related receptor gamma (ERRγ) and G-protein-coupled receptor 30 (GPR30) by affecting the reproductive, metabolic and immune systems, along with neurodevelopmental processes. BPA regulates gene expression by activating intracellular transduction pathways via the cell surface and nuclear receptors, and also affects the antioxidant system (ROS accumulation) and impairs mitochondrial function. Human exposure to BPA occurs following BPA migration from the above-mentioned product containers and devices to the environment, including food; this type of migration represents the main route of exposure. For several years, the European Food Safety Authority (EFSA) has evaluated and updated the information on the effects of BPA on human health, including reduced fertility, an increased risk of cancer and the development of metabolic and cardiovascular diseases. Recently, the EFSA has established a new Tolerable Daily Intake value based on the observed immunological effects of exposure to BPA, and has identified the immune system as that most sensitive to BPA exposure. To reduce these health risks, the use and production of BPA have been restricted. However, BPA has been replaced by several bisphenol analogs, such as bisphenol S (BPS) and bisphenol F (BPF); their toxicological effects are comparable to those of BPA, and they have recently been detected in the environment, in food and even in humans. Owing to the rapidly expanding uses of bisphenol analogs, increasing attention has been paid to their toxicity and environmental effects.

The purpose of this Special Issue, entitled “Molecular Mechanisms of Bisphenol A Toxicity and Effects of Environmental Levels on Health”, is to present studies on the molecular mechanism not only of Bisphenol A, but also of its analogues, including BPS, BPF and bisphenol AF (BPAF).

Qian, L. et al. [1] observed that BPS, BPF and BPAF induce toxic and estrogenic effects similar to those of BPA, affecting the growth and reproduction of Daphnia magna through growth inhibition, oxidative stress and altered gene expression. Similarly, in an in vitro study on bovine granulosa cells, the authors observed epigenetic alterations induced by BPA involving the female reproductive tract [2]. Specifically, the results of this study showed that BPA can induce apoptosis through a pathway that does not involve miR-21 signaling, but under specific conditions, it may act through miR-21 and programmed cell death 4 (PDCD4) to induce antiapoptotic and pro-oncogenic effects in cells.

In addition to the toxic effects of BPA and its analogues on growth and reproduction, Liu, R et al. [3] present interesting and novel data on the mechanisms underlying the hepatotoxicity and intestinal dysbiosis induced by BPA. Notably, they observed that in Sprague Dawley rats, BPA induced a radical alteration in their microbiota, characterized by an increase in the ratio of Firmicutes to Bacteroidetes (F/B) and a relative abundance of Proteobacteria in feces; meanwhile, it decreased the relative abundance of Prevotella_9 and Ruminococcaceae_UCG-014, which was positively correlated with short-chain fatty acid (SCFA) content.

Oxidative stress induction was observed in an in vitro study on erythrocytes, where BPA and three intermediate products of its degradation, namely phenol, hydroquinone and 4-isopropylphenol, increased the reactive oxygen and nitrogen species (RONS) levels and reduced glutathione (GSH) levels [4].

The valuable contributions included in the present update shed light on several mechanisms of action of BPA, as well as its analogues and degradation products; however, further studies are needed on the mechanisms of toxicity and effects exerted by bisphenol analogs that take into account combined exposure.

## References

[1] Qian L., Chen C., Guo L., Deng J., Zhang X., Zheng J., Wang G., Zhang X. (2022). Developmental and Reproductive Impacts of Four Bisphenols in Daphnia magna. Int. J. Mol. Sci..

[2] Sabry R., Williams M., Werry N., LaMarre J., Favetta L.A. (2022). BPA Decreases PDCD4 in Bovine Granulosa Cells Independently of miR-21 Inhibition. Int. J. Mol. Sci..

[3] Liu R., Liu B., Tian L., Jiang X., Li X., Cai D., Sun J., Bai W., Jin Y. (2022). Exposure to Bisphenol A Caused Hepatoxicity and Intestinal Flora Disorder in Rats. Int. J. Mol. Sci..

[4] Makarova K., Olchowik-Grabarek E., Drabikowski K., Kurkowiak J., Zawada K. (2023). Products of Bisphenol A Degradation Induce Cytotoxicity in Human Erythrocytes (In Vitro). Int. J. Mol. Sci..

